# MicroRNA Expression in Response to Controlled Exposure to Diesel Exhaust: Attenuation by the Antioxidant *N*-Acetylcysteine in a Randomized Crossover Study

**DOI:** 10.1289/ehp.1205963

**Published:** 2013-04-12

**Authors:** Masatsugu Yamamoto, Amrit Singh, Francesco Sava, Mandy Pui, Scott J. Tebbutt, Christopher Carlsten

**Affiliations:** 1 Department of Medicine, Division of Respiratory Medicine, University of British Columbia, Vancouver, British Columbia, Canada; 2 James Hogg Research Centre, Institute for HEART + LUNG Health, St. Paul’s Hospital, University of British Columbia, Vancouver, British Columbia, Canada; 3 Vancouver Coastal Health Research Institute, Vancouver General Hospital, Vancouver, British Columbia, Canada; 4 Networks of Centres of Excellence for Commercialization and Research (NCE CECR) Prevention of Organ Failure (PROOF) Centre of Excellence, Vancouver, British Columbia, Canada

**Keywords:** air pollution, asthma, controlled diesel exhaust exposure, hsa-miR-144, microRNA, *N*-acetylcysteine, NanoString nCounter assay, *NRF2*, oxidative stress, peripheral blood

## Abstract

Background: Adverse health effects associated with diesel exhaust (DE) are thought to be mediated in part by oxidative stress, but the detailed mechanisms are largely unknown. MicroRNAs (miRNAs) regulate gene expression post-transcriptionally and may respond to exposures such as DE.

Objectives: We profiled peripheral blood cellular miRNAs in participants with mild asthma who were exposed to controlled DE with and without antioxidant supplementation.

Methods: Thirteen participants with asthma underwent controlled inhalation of filtered air and DE in a double-blinded, randomized crossover study of three conditions: *a*) DE plus placebo (DEP), *b*) filtered air plus placebo (FAP), or *c*) DE with *N*-acetylcysteine supplementation (DEN). Total cellular RNA was extracted from blood drawn before exposure and 6 hr after exposure for miRNA profiling by the NanoString nCounter assay. MiRNAs significantly associated with DEP exposure and a predicted target [nuclear factor (erythroid-derived 2)-like 2 (*NRF2*)] as well as antioxidant enzyme genes were assessed by reverse transcription–quantitative polymerase chain reaction (RT-qPCR) for validation, and we also assessed the ability of *N*-acetylcysteine supplementation to block the effect of DE on these specific miRNAs. 8-hydroxy-2´-deoxyguanosine (8-OHdG) was measured in plasma as a systemic oxidative stress marker.

Results: Expression of miR-21, miR-30e, miR-215, and miR-144 was significantly associated with DEP. The change in miR-144 was validated by RT-qPCR. *NRF2* and its downstream antioxidant genes [glutamate cysteine ligase catalytic subunit (*GCLC*) and NAD(P)H:quinone oxidoreductase 1 (*NQO1*)] were negatively associated with miR-144 levels. Increases in miR-144 and miR-21 were associated with plasma 8-hydroxydeoxyguanosine 8-OHdG level and were blunted by antioxidant (i.e, DEN).

Conclusions: Systemic miRNAs with plausible biological function are altered by acute moderate-dose DE exposure. Oxidative stress appears to mediate DE-associated changes in miR-144.

Epidemiological and clinical studies have shown that exposure to air pollutants is associated with increased mortality, higher hospital admission rates for respiratory and cardiovascular diseases and cancer, as well as decreased physiological function such as lung function in patients with lung disease (Peng et al. 2009; Pope et al. 1995, 2011; Sava and Carlsten 2012; Zanobetti et al. 2000). Asthma is characterized by chronic inflammation and airway hyperresponsiveness. Air pollutants including combustion-derived particulate matter (PM) have been linked to asthma symptoms and exacerbations (Atkinson et al. 2001; Friedman et al. 2001; Guo et al. 1999; von Klot et al. 2002). Diesel exhaust (DE) is a key source of ambient PM < 2.5 µm in aerodynamic diameter (PM_2.5_), which penetrates deeply into the lungs and has been associated with acute electrocardiographic changes and worsening of asthmatic lung function, as shown in both experimental (Mills et al. 2007) and epidemiological studies (McCreanor et al. 2007). Moreover, systemic pathways have been suggested to contribute to the adverse health effects of air pollution (Brook et al. 2010). A number of investigators have pointed to the importance of antioxidant pathways in the effects of air pollution on asthma. NAD(P)H:quinone oxidoreductase 1 (*NQO1*), which has a role in oxidative stress, may be related to asthma susceptibility among persons exposed to traffic-related air pollution (Castro-Giner et al. 2009). The oxidative stress response to DE has been demonstrated in a controlled human exposure model (Behndig et al. 2006). Antioxidant responses to oxidative stress, including those within the nuclear factor (erythroid-derived 2)-like 2 (*NRF2*) pathway, such as *NQO1* and glutamate cysteine ligase catalytic subunit (*GCLC*), play essential roles in response to injury caused by environmental oxidants such as hyperoxia, DE particles, and cigarette smoke (Cho et al. 2006). However, the mechanism of the DE-related pathophysiology that mediates dysregulation of such antioxidant mechanisms in humans has not been fully elucidated.

MicroRNAs (miRNAs) are a class of small noncoding RNAs with a posttranscriptional regulatory function on gene expression, whereby they exert control over processes such as cellular proliferation, apoptosis, and differentiation (He and Hannon 2004). MiRNAs are important mediators of biological functions and their dysregulation has been implicated in a wide range of diseases, including malignancies, heart diseases, inflammation, and lung diseases (Holloway et al. 2012; Oglesby et al. 2010; Small and Olson 2011). Exposure to air pollutants in particular has the potential to dysregulate miRNA (Jardim 2011) and this may be reflected systemically, as shown in peripheral blood mononuclear cells in steel plant workers exposed to PM (Bollati et al. 2010). MiRNAs were suggested to play roles in allergic airway inflammation, according to an experimental model (Lu et al. 2009), although a study comparing miRNA profiles in airway biopsies revealed no significant expression differences across 227 miRNAs in participants with mild asthma compared with healthy volunteers (Williams et al. 2009). Recently, we clarified that miR-192 in peripheral blood was altered after exposure to inhaled allergen in human asthmatics (Yamamoto et al. 2012). Therefore, one may postulate that miRNA profiles altered by exposure may be involved in pathophysiological changes in asthmatics, but there have been no studies that have directly evaluated miRNA changes after controlled acute exposure of participants with asthma to air pollutants.

Our *in vivo* human model, using freshly generated exhaust that is diluted and aged to reflect real-world conditions, allows us to evaluate mechanisms of DE exposure on asthma biology (Birger et al. 2011; Saxon and Diaz-Sanchez 2005). In the present study, we hypothesized that controlled DE exposure alters miRNA profiles in peripheral blood cells and that changes in specific miRNAs will be attenuated by antioxidant supplementation.

## Materials and Methods

We conducted a randomized crossover, double-blind experiment with each participant exposed on 3 different days to each of three conditions: filtered air exposure plus placebo (FAP), DE exposure (300 µg PM_2.5_/m^3^) plus placebo (DEP), and DE exposure plus *N*-acetylcysteine (NAC) supplementation (DEN). We selected a DE concentration of 300 µg PM_2.5_/m^3^ because it reflects common occupational and intermittent high ambient concentrations and is aligned with most human studies in the literature (Behndig et al. 2006; Crüts et al. 2008; Hussain et al. 2012).

Each exposure was for 2 hr and each associated treatment (placebo or 600 mg NAC as antioxidant) was in identical-appearing capsule form, taken orally three times per day, for 5 days preceding and also on the day of exposure (total of 6 days). The order of exposure was randomized. Each participant was exposed to each condition and there was a minimum 2-week washout period between conditions. This study was approved by the Clinical Research Ethics Board of the University of British Columbia and all study participants provided written informed consent upon enrolling in the study.

*Participants*. Thirteen participants were recruited at the Air Pollution Exposure Laboratory (APEL) in Vancouver, British Columbia, Canada. Participants were 19- to 35-year-old nonsmokers who had had physician-diagnosed asthma for at least 1 year and/or a methacholine challenge with ≤ 8 mg/mL in terms of the PC_20_, a provocative concentration of methacholine that induces a 20% fall in forced expiratory volume in 1 sec (FEV_1_). All participants were stable in terms of asthma symptoms [assessed by the asthma control questionnaire (Juniper et al. 1999)] and were free of respiratory infections for 4 weeks prior to and during the study period. The participants were free from current use of inhaled corticosteroids, regular use of bronchodilator, and use of vitamin A, C, or E supplements. Throughout the study, participants were asked to withhold long-acting β2-agonists 48 hr prior to spirometry, short-acting β2-agonists 8 hr prior to spirometry, and caffeine 4 hr prior to methacholine challenges. The participants maintained a stable diet, including intake of cruciferous vegetables, over the course of the study; thus, in the context of the crossover design, diet was not considered confounding.

*Methacholine challenge for airway hyperresponsiveness*. Spirometry and methacholine challenge were performed according to the American Thoracic Society standards (Crapo et al. 2000; Miller et al. 2005) 18 hr preexposure and 30 hr postexposure. PC_20_ was determined by interpolation of the logarithmic (base 2) concentration–response curve obtained using the 2-min tidal breathing method. Airway responsiveness to methacholine was quantified by calculating the dose–response slope (DRS) (de Meer et al. 2005).

*Controlled DE exposure*. Controlled DE exposures were conducted as reported previously (Birger et al. 2011). Briefly, a 6.0-kW diesel generator was operated under discrete loads to simulate diesel on-road emission. A portion of raw exhaust was drawn into the primary dilution system with compressed air at standard temperature (20°C) and relative humidity (40%) levels. The exhaust was further diluted by high efficiency particulate air–filtered air (FA) and then aged for 4 min before entering the 4 ft × 6 ft × 7 ft exposure booth. An average concentration of DE was maintained at 301 µg PM_2.5_/m^3^ with an average SD of 16 µg/m^3^. During exposures, participants alternated between rest and light exercise (15 min/hr) on a stationary bicycle. The load of the exercise was set to achieve a minute ventilation of 15 L/min/m^2^ body surface area.

*Blood collection and RNA extraction*. Peripheral blood was collected preexposure, and 6 hr after the beginning of exposure, into PAXgene Blood RNA tubes (PreAnalytiX, Qiagen/BD, Valencia, CA, USA) and EDTA tubes for complete blood cell counts, leukocyte differentials, and CD3^+^ T-cell counts [see Supplemental Material, p. 3 and Figure S1A (http://dx.doi.org/10.1289/ehp.1205963)]. All of the collected samples were frozen and transported to the laboratory on dry ice, where they were stored at –80°C until RNA extraction. From thawed PAXgene tube samples, total RNA was purified from 2.5 mL of whole blood according to the manufacturer’s protocols using the PAXgene blood miRNA kit (Qiagen, Chatsworth, CA, USA). The quantity and quality of RNA were assessed by NanoDrop 8000 Spectrophotometer (Thermo Scientific, Wilmington, DE, USA) and Agilent 2100 Bioanalyzer following the RNA 6000 Nano Kit protocol (Agilent Technologies, Santa Clara, CA, USA). The RNA samples were then stored at –80°C before nCounter analyses.

*NanoString nCounter assay*. Holistic assay for miRNA expression was performed using nCounter® miRNA Expression Assay Kits (NanoString Technologies, Seattle, WA, USA) at NanoString Technologies. This method enables multiplexed direct digital counting of RNA molecules (Geiss et al. 2008). To prepare miRNA molecules for hybridization in the nCounter assay, proprietary DNA sequences called miRtags were ligated to the mature miRNAs using bridging oligonucleotides (bridges). After ligation and purification, the tagged mature miRNAs were then hybridized to color-coded reporter probes and biotinylated capture probes. The capture probe allows the complex to be immobilized for data collection by measurement of its color code. A total of 734 human and human-associated viral miRNAs were simultaneously assayed. All assays were performed using 100 ng of total RNA following the standard nCounter miRNA Assay Protocol. Hybridizations were carried out by mixing 5 µL of each miRNA multiplex assay with 20 µL NanoString nCounter reporter probe mix and 5 µL capture probe mix (30 µL total reaction volume), and incubating the hybridizations at 65°C for 18 hr. To assess technical reproducibility, two samples were assayed in triplicate. The data set was preprocessed [see Supplemental Material, p. 3 (http://dx.doi.org/10.1289/ehp.1205963)] then normalized to the sum of the miRNAs detected above background across all 52 samples (4 samples each from 13 participants).

*Reverse transcription–quantitative polymerase chain reaction (RT-qPCR)*. RT-qPCR for miRNAs and gene expressions (*NRF2*, *GCLC*, *NQO1*) was carried out using TaqMan MiRNA Assays and TaqMan Gene Expression Assay (Applied Biosystems, Foster City, CA) according to the manufacturer’s protocol [see Supplemental Material, p. 4 (http://dx.doi.org/10.1289/ehp.1205963)]. Cycle threshold (Ct) values were calculated with the SDS software (Applied Biosystems) using automatic baseline settings. Ct values > 35 were considered to be below the detection level of the assay. *RNU44* (small nucleolar RNA, C/D box 44) and β-glucuronidase were used for normalizing the expression level of selected miRNAs and gene expressions, respectively. The median Ct value was subtracted from the corresponding Ct value, resulting in relative quantification of miRNA expression using the 2^–ΔΔCt^ method (Livak and Schmittgen 2001).

*Measurement of oxidative stress marker*. The plasma samples were analyzed for 8-hydroxy-2´-deoxyguanosine (8-OHdG) levels using an OxiSelect oxidative DNA damage ELISA kit (Cell Biolabs, San Diego, CA, USA) according to the manufacturer’s protocol. The absorbance was read at 450 nm using a microplate reader. The 8-OHdG levels were calculated and expressed in nanograms per milliliter.

*Data processing and statistical analysis*. Data were analyzed in R, version 2.12.1 (R Foundation for Statistical Computing, Vienna, Austria). We adopted log2-transformed data in the series of analyses for NanoString code counts, RT-qPCR expression levels and oxidative stress marker (i.e., 8-OHdG) to satisfy the assumption of normality in linear models. Moderated robust regression in the Linear Model for Microarrays (limma) library (Smyth 2004) from Bioconductor (Gentleman et al. 2004) was used to determine miRNAs that were significantly associated with the exposure condition, incorporating each participant as a factor. A Benjamini–Hochberg false discovery rate (FDR) of < 0.05 was considered significant. Associations of three target gene expressions with miR-144 were analyzed on RT-qPCR data using limma, adjusted for exposure conditions with the incorporation of each participant as a factor. Association of 8-OHdG level with miR-144 was analyzed using a linear mixed effect model (nlme library), adjusted with NAC supplementation as a fixed effect and with participants as a random effect, confined to the data on DE exposure (DEP and DEN). The association of DRS with miR-144 or *NRF2* levels was analyzed using a linear mixed effect model adjusted with exposure conditions as fixed effects and with participants as a random effect. For comparison of replicate data and evaluating the technical replicability of RT-qPCR, Pearson correlation coefficients (*r*) were calculated. Complete blood cell counts, leukocyte differentials, and CD3^+^ T-cell counts were analyzed using analysis of variance or paired Student’s *t*-test. A *p*-value of < 0.05 was considered significant.

## Results

*Participants*. A total of 13 participants were assessed in the study. Baseline characteristics are presented in Supplemental Material, Table S1 (http://dx.doi.org/10.1289/ehp.1205963). The participants were 11 Caucasians and 2 Asians. All participants’ blood cell counts were within normal limits. Because complete blood cell counts and white blood cell differentials (see Supplemental Material, Table S2) were not significantly different among six sample groups (those obtained before and after each of three conditions) analyzed in this study, we did not adjust for cell type in further analyses.

*Differentially expressed miRNA in NanoString nCounter assay*. Eighty-one of the 734 miRNAs assayed were above background across all 52 samples. For determining miRNAs differentially expressed by FAP versus DEP, first the difference between pre- and postexposure log2-transformed code counts for each condition and each miRNA was computed (designated “delta”). Linear models for microarrays (limma) determined that miR-320a expression was significantly different between delta FAP and delta DEP at a FDR of 0.05 [see Supplemental Material, Table S3, Figure S2 (http://dx.doi.org/10.1289/ehp.1205963)], with the delta DEP lower than delta FAP indicating a relative decrease in miR-320a upon DE exposure as compared with FA exposure.

Next, miR-30e, miR-144, miR-21, and miR-215 were significantly differentiated between post-FAP and post-DEP at an FDR of 0.05 using limma [see Supplemental Material, Table S4, Figure S3 (http://dx.doi.org/10.1289/ehp.1205963)]. Each of these miRNAs was higher post-DEP than post-FAP. None of these miRNAs were significantly different between pre-FAP and pre-DEP at an FDR of 0.05. The order of exposure (exposed first to FA, or first to DE), which was treated as a fixed effect with a linear mixed effect model, was not associated with any of these miRNAs (data not shown).

*Reproducibility of the NanoString technology*. For evaluating the reproducibility with replicate data, two RNA samples were assayed in triplicate. To test for reproducibility, the code count data in triplicate sample set was compared for 81 miRNAs that were assessed in all sample sets as well as all 734 miRNAs measured in the nCounter assay. Although the scatter plot showed dispersion of the raw code counts for miRNAs with low expression level, the correlation of the technical replicates for preprocessed 81 miRNAs with higher expression level was strong, showing excellent reproducibility of the assay [see Supplemental Material, Figure S4 (http://dx.doi.org/10.1289/ehp.1205963)].

*Technical validation using RT-qPCR*. From the results of differentially expressed miRNAs, we selected five miRNAs (miR-320a, miR-21, miR-30e, miR-144, miR-215) for technical validation using RT-qPCR. Normalized expression data for these five miRNAs were compared between the two assays. The correlation coefficient was highest for miR-144 (*r* = 0.82, *p* < 0.0001), which showed the highest nCounter code count, whereas the correlation coefficient was lowest for miR-215 (*r* = 0.34, *p* 0.09), which showed the lowest nCounter code count [see Supplemental Material, Figure S5 (http://dx.doi.org/10.1289/ehp.1205963)].

**Figure 1:**
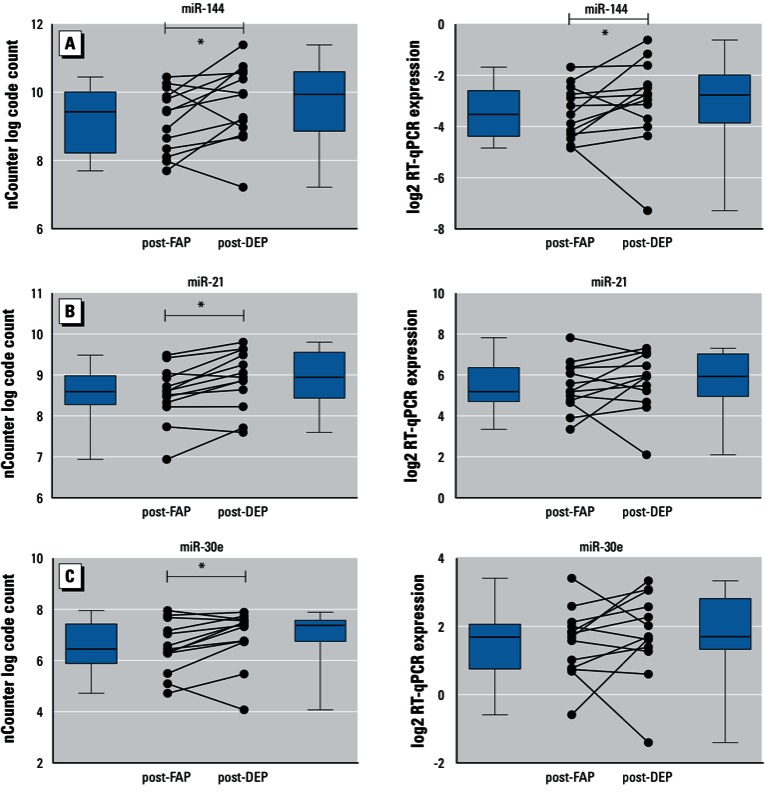
The normalized nCounter data (left panels) and the expression levels measured with RT-qPCR (right panels) of miR-144 (*A*), miR-21 (*B*), and miR-30e (*C*) are shown. Dot plots show 13 individual participants’ data, boxes delineate first and third quartile, whiskers represent minima and maxima respectively, solid lines within boxes indicate medians. *FDR < 0.05.

The results of the nCounter assay comparing post-FAP and DEP for miR-144 were validated at an FDR of 0.05 using RT-qPCR. The other miRNAs did not reach statistical significance, although post comparisons for miR-21 and miR-30e demonstrated marginal statistical significance (FDR 0.058 for one-sided test in each case) [Table 1 and Figure 1, also see Supplemental Material, Figure S6 (http://dx.doi.org/10.1289/ehp.1205963)].

**Table 1:** Validation with RT-qPCR comparing post-FAP and post-DEP (one-sided test).

miRNA	log2 fold change^*a*^	*t-*Value	*p-*Value	FDR
hsa-miR-144	0.58	3.09	0.002	0.007
hsa-miR-30e	0.34	1.89	0.032	0.058
hsa-miR-21	0.32	1.75	0.043	0.058
hsa-miR-215	0.06	0.33	0.37	0.37
^***a***^Positive log2 fold change indicates higher expression after DEP relative to FAP.				

*CD3^+^ T-cell counts*. We counted CD3^+^ T cells to clarify whether the miR-144 increase in post-DEP was attributable to a change in T cells, given a report that miR-144*, which derives from the same primary miRNA as miR-144, was highly expressed in T cells (Liu et al. 2011). The CD3^+^ T-cell counts did not differ significantly between post-FAP and post-DEP [see Supplemental Material, Figure S1B (http://dx.doi.org/10.1289/ehp.1205963)].

**Figure 2:**
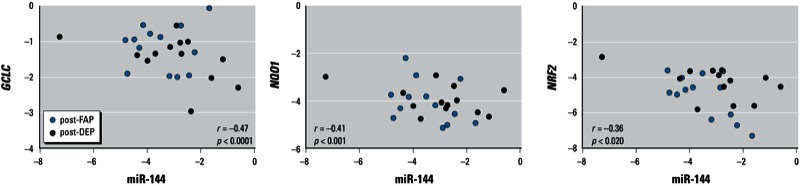
Scatter plots of miR-144 compared with *GCLC*, *NQO1*, and *NRF2*. The plots show log2-transformed levels measured by RT-qPCR in post-FAP and post-DEP. The *p*-values refer to the association analyzed using limma.

*The association of* NRF2 *and antioxidant genes with miR-144*. Among predicted target genes from a database of miRNAs (http://www.miRDB.org), we selected *NRF2* as a candidate target gene for miR-144, whose role in oxidative stress pathways was previously demonstrated (Sangokoya et al. 2010). Given the purported role of miR-144 in target gene expression, expression of genes coding for NRF2 and enzymes NQO1 and GCLC, which are downstream of *NRF2*, were assessed and found negatively associated with miR-144 levels (*p* = 0.020, 0.001, < 0.0001, respectively, Figure 2).

**Figure 3:**
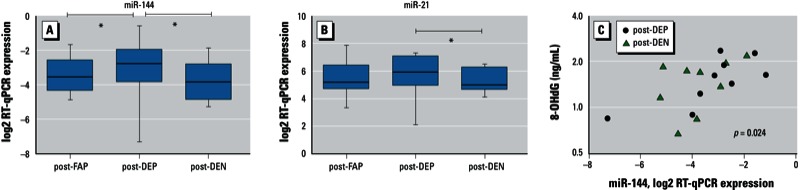
The effect of antioxidant on miRNAs and association with oxidative stress. (*A,B*) RT-qPCR plots of two miRNAs for three exposures. The normalized RT-qPCR data comparing FAP, DEP, and DEN are shown for miR-144 (*A*) and miR-21 (*B*). (*C*) Scatter plots of miR-144 compared with 8-OHdG of post-DEP and post-DEN; the *x*-axis represents log2-transformed expression, and the *y*-axis represents plasma 8-OHdG level on log2 axis. The *p*-value refers to the association analyzed with a linear mixed effect model. *FDR < 0.05.

*Comparison between post-DEP and post-DEN*. To evaluate the preventive effects of NAC on the altered level of differentially expressed miRNAs, we selected three miRNAs successfully or marginally validated between FAP and DEP (i.e., miR-144, miR-21, miR-30e) for comparison of the expression levels post-DEN and post-DEP, among those nine participants who provided sufficient blood under the DEN condition for analysis. These nine participants did not significantly differ from the four participants with insufficient blood, in terms of sex, age, BMI, or FEV_1_. MiR-144 and miR-21 were significantly decreased post-DEN relative to post-DEP [Table 2, Figure 3A,B; see also Supplemental Material, Figure S7 (http://dx.doi.org/10.1289/ehp.1205963)].

**Table 2:** Comparison of RT-qPCR expression data comparing post-DEP and post-DEN (two-sided test)

miRNA	log2 fold change^*a*^	*t-*Value	*p-*Value	FDR
hsa-miR-144	–0.81	–3.79	0.0014	0.0043
hsa-miR-21	–0.48	–2.46	0.025	0.038
hsa-miR-30e	–0.03	–0.22	0.83	0.83
^***a***^Negative log2 fold change indicates lower expression after DEN relative to DEP.				

*Association of miR-144 regulated by NAC with 8-OHdG*. To evaluate the relationship between systemic oxidative stress and miR-144, we analyzed the association of miR-144 level post-DEP and post-DEN with plasma 8-OHdG, using a linear mixed effect model. Relative expression level of miR-144 was positively associated with log2-transformed 8-OHdG level with fixed effect estimates of 1.58 (95% CI: 0.40, 2.77; *p* = 0.024) (Figure 3C).

*Association of miR-144 and* NRF2 *with airway responsiveness*. To evaluate the relationship of airway responsiveness with miR-144 as well as *NRF2* levels, we analyzed the association with DRS measured postexposure using a linear mixed effect model. Significant associations were not observed with miR-144 or *NRF2*.

## Discussion

To our knowledge, we have demonstrated for the first time alterations in systemic miRNA profiles associated with acute DE exposure in humans as well as oxidative stress, using a controlled exposure and a randomized crossover design. The high sensitivity of the nCounter assay allowed for novel elucidation of miRNAs that had been significantly altered by DE exposure. Interestingly, the increase in hsa-miR-144 associated with inhalation of DE was prevented by antioxidant supplementation. Additionally, negative associations of miR-144 with expression of *NRF2* and downstream enzymes suggest a biologically plausible mechanism in response to systemic oxidative stress in DE exposure; a previous report also suggests that miR-144 targets *NRF2*, which is a transcription factor regulating the cellular responses to oxidative stress (Sangokoya et al. 2010). *NRF2* binds to antioxidant response elements in the upstream regions of target genes and subsequently activates gene transcription for the antioxidant enzymes such as NQO1 and GCLC, so that *NRF2* activation determines an adaptive response to the exposure to oxidant pollutants and provides a pivotal defense mechanism against air pollutants (Lodovici and Bigagli 2011). Given the low mRNA expression level and the role of transcription factor in gene expression, *NRF2* function would be better assessed in future studies using an activity assay to more definitively show that *NRF2* function is directly regulated by miR-144. However, our data linking miR-144 to an oxidative exposure (i.e., DE) and genes known to be important within the oxidative stress pathway (*NRF2*, *GCLC*, and *NQO1*) is buttressed by our having shown both a preventive effect of NAC on up-regulated miR-144 and also a positive association with the systemic oxidative stress marker 8-OHdG.

NAC is a thiol antioxidant compound and a glutathione precursor, effectively acting as reactive oxygen species scavenger (Rhoden et al. 2004). The protective effects of NAC on the toxic effects of particulate air pollution has been reported using *in vivo* model of inhalation exposure to PM in a rat model, showing that NAC treatment protected lung tissue exposed to PM from oxidative stress as well as inflammatory changes. An animal study using a mouse model exposed to phosgene for a lung injury model reported that NAC administration elevated the *NRF2* expression levels in the lung, even 3 hr after exposure. They showed that the preventive mechanism of NAC might be mediated by the synthesis of reduced glutathione by up-regulating the *NRF2*/glutathione reductase pathway. An experimental model using rat lungs revealed that environmental cigarette smoke caused miRNA perturbation and that NAC exerted preventive effects therein (Izzotti et al. 2010). It did not report involvement of miR-144, although they suggest that the response to oxidative stress induced by environmental exposures may perturb miRNA profiles *in vivo*. MiRNAs would not likely respond in common in the blood and lung, given the evidence for tissue-specific miRNA profiles (Liang et al. 2007). *NRF2* is widely expressed in blood cells, including various types of immune cells, and is important for maintaining intracellular glutathione levels and, consequently, redox homeostasis. Future studies investigating lung and airway specimens in parallel for changes in miRNAs are warranted to further understand the miRNA-mediated mechanism of health effects due to air pollution.

Although the estimated effect of DE on miR-21 was only marginally significant, our results showing evidence of preventive effects of NAC on miR-21 suggest a possible relationship between miR-21 and oxidative stress due to DE exposure. MiR-21 has been associated with many pathophysiological pathways including inflammation, oxidative stress, atherosclerosis, and carcinogenesis (Kuehbacher et al. 2008; Nana-Sinkam et al. 2009). MiR-21 has been reported to be up-regulated in asthma models (Lu et al. 2009; Mattes et al. 2009) as well as in response to oxidative stress in various experimental models (Bollati et al. 2010; Chau et al. 2012; Cheng et al. 2009; Lin et al. 2009), supporting the evidence that miR-21 plays a role in allergic inflammation and oxidative stress. The toll-like receptor pathways, which are also activated by PM, are also regulated by miR-21 (Lu et al. 2011; Sheedy et al. 2010; Shoenfelt et al. 2009), suggesting that miRNAs are involved in the alteration of innate immunity caused by DE. Given these reports showing its substantial role in immune and inflammatory response, further studies focusing on the role of miR-21 in response to air pollution may be informative.

In our experiments to perform technical validation with RT-qPCR, the results from the nCounter assay were not validated for all miRNAs assayed in RT-qPCR; only miR-144 validated in terms of strict statistical significance despite our finding that technical replicability for the nCounter assay was excellent. The NanoString nCounter assay, which has been utilized also for mRNAs, is a novel method that enables digital counting of individual RNAs in a single reaction without the need for amplification differently from RT-qPCR (Geiss et al. 2008). The fold changes detected in blood samples were smaller than in organ tissue samples. To detect the significant changes, higher expression values in the nCounter assay resulted in successful validation (miR-144) or marginally significant validation (miR-21, miR-30e), whereas expression below ~ 100 (log code count 6.64) did not (miR-320a, miR-215). Such possible difference in sensitivity between nCounter assay and RT-qPCR for miRNAs should be further explored as the various technologies are studied in a variety of pertinent clinical research contexts.

A limitation of our study is that we performed only technical validation, but not biological validation, because we did not have the appropriate substrate for functional assays; future studies assessing target gene expression are desirable. A further goal of future work would be to determine the physiological correlates of these changes in miRNA; several studies using experimental models of allergic airway inflammation reported the functions of miRNAs in airway hyperresponsiveness as well as airway inflammation (Lu et al. 2009; Mattes et al. 2009), but we could not detect a significant association of miRNA or target gene levels with airway hyperresponsiveness. This may not be surprising given the complexity of factors determining airway responsiveness and that functional changes in the airway likely reflect local, tissue-specific changes that were not investigated here but are a worthy topic for future investigation.

## Conclusions

MiRNA profiles in peripheral blood can change within hours of acute DE exposure. MiR-144 was up-regulated by DE and was associated with systemic oxidative stress; the DE-related increase in miR-144 was attenuated by NAC supplementation. The *NRF2* pathway may be regulated by miR-144 because changes in miR-144 were associated with expression of *NRF2*, *GCLC*, and *NQO1*.

## Supplemental Material

(1.2 MB) PDF
