# Supplementary material for: MicroRNA Expression in Response to Controlled Exposure to Diesel Exhaust: Attenuation by the Antioxidant N-Acetylcysteine in a Randomized Crossover Study
Source: Environ Health Perspect. 2013 Apr 12;121(6):670–5. doi: 10.1289/ehp.1205963 (PMC3672916; doi:10.1289/ehp.1205963)
Supplement: (1.2 MB) PDF [file ehp.1205963.s001.pdf]

Environmental Health Perspectives doi:10.1289/ehp.1205963

Supplemental Material

MicroRNA Expression in Response to Controlled Exposure to Diesel Exhaust: Attenuation by the Antioxidant n-Acetylcysteine in a Randomized Crossover Study

Masatsugu Yamamoto, Amrit Singh, Francesco Sava, Mandy Pui, Scott J. Tebbutt, and Christopher Carlsten

Table of Contents

Methods

CD3 positive T cells 3

Preprocessing of nCounter miRNA Codeset 3

RT-qPCR 4

Tables

Table S1. Participant characteristics 5

Table S2. Complete blood cell count, including white blood cell differential, before and 6 hours after each condition 6

Table S3. Comparison of nCounter data between delta-FAP and delta-DEP 7

Table S4. Comparison of nCounter data between post-FAP and post-DEP 8

Figures

Figure S1. CD3 positive T cell counts 9

Figure S2. Comparison of nCounter data between delta-FAP and delta-DEP 10

Figure S3. Comparison of nCounter data between post-FAP and post-DEP 11

Figure S4. Scatter plots of technical replicates for nCounter assay 12

Figure S5. Scatter plots of candidate miRNAs comparing nCounter assay and RT-qPCR 13

Figure S6. Validation of miRNA levels with RT-qPCR between post-FAP and post-DEP 14

Figure S7. Comparison of miRNA level between post-DEP and post-DEN 15

Supplemental Material, Methods

CD3 positive T cells

Blood was collected into EDTA tubes at 6 hours post-exposure. Whole blood was incubated with fluorescent antibodies to human CD3 and CD45 (BD Biosciences, San Jose, CA) at room temperature for 30 minutes. A lysing solution (BD Biosciences) was added to remove red blood cells. Cells were washed and resuspended in PBS with 0.1% paraformaldehyde. Flow cytometry was performed using a FACSCanto flow cytometer (BD Biosciences) to be analysed as in Supplemental Material, Figure S1A. Cellular debris was eliminated on the SSC/FSC scattergram. Leukocytes were identified as expressing CD45. T lymphocytes were identified as expressing CD45 and CD3. The percentage of T lymphocytes out of the $CD45^+$ cells (leukocytes) was calculated for each participant, post DEP and FAP.

Preprocessing of nCounter miRNA Codeset

The nCounter assay for each sample consisted of six positive controls (0.125-128 fM), eight negative controls, five control mRNAs (ACTB, B2M, GAPDH, RPL19 and RPLP0) and 734 miRNAs. Prior to normalization, several probes in the codeset required background subtraction. The probes for which the background subtraction calculation produced a negative number were set to 1. To account for slight differences in assay efficiency (hybridization, purification, and binding) the data were initially normalized to the sum of 6 positive RNA spike-in controls. For each sample, the mean plus 2 times the standard deviation of the 8 negative controls was subtracted from each miRNA count in that sample. Only miRNAs with non-negative counts across all samples were retained for downstream analysis.

RT-qPCR

RT-qPCR for miRNAs was carried out using TaqMan MiRNA Assays (Applied Biosystems, Foster City, CA) according to the manufacturer's instruction. The TaqMan MicroRNA Reverse Transcription Kit (Applied Biosystems) was used for the preparation of cDNA. Reverse transcription reactions were performed in a volume of 15 μl, and each reaction contained 10 ng of total RNA including miRNA. The PCR reaction mix consisted of the RT product, TaqMan 2X Universal PCR Master Mix and the appropriate 5X MicroRNA Assay Mix containing primers and probe for the miRNA of interest. The gene expressions (NRF2, GCLC, NQO1) were measured using TaqMan Gene Expression Assay (Applied Biosystems) after RNA in samples was converted into cDNA using SuperScript II Reverse Transcriptase (Invitrogen, Carlsbad, CA, USA) according to the manufacturer's instructions. All TaqMan assays were run in triplicate on an ABI Prism 7900 or ViiA7, applying 40 PCR cycles. Cycle threshold (Ct) values were calculated with the SDS software (Applied Biosystems) using automatic baseline settings. Ct values >35 were considered to be below the detection level of the assay.

Supplemental Material, Table S1. Participant characteristics.

Participant	Sex	Age (years)	Body mass index (kg/m^2)	FEV_1 (%predicted)	Asthma diagnosis
1	F	28	25.1	97.0	yes
2	M	26	20.8	102.0	yes
3	F	33	21.3	120.0	no
4	M	30	30.7	82.0	yes
5	M	25	25.6	107.0	yes
6	M	33	24.9	86.0	yes
7	F	29	24.8	106.0	no
8	F	25	21.7	86.0	yes
9	M	34	24.2	66.0	yes
10	F	35	23.1	68.0	yes
11	M	19	24.4	79.0	yes
12	F	24	24.5	93.0	yes
13	M	26	28.7	96.0	yes

FEV_1: forced expiratory volume in one second

Supplemental Material, Table S2. Complete blood cell count, including white blood cell differential, before and 6 hours after each condition

Cell type	pre-FAP	post-FAP	pre-DEP	post-DEP	pre-DEN	post-DEN	p value
Red blood cell (x 10^6/μl)	4.64 ± 0.35	4.59 ± 0.47	4.67 ± 0.41	4.65 ± 0.47	4.60 ± 0.36	4.61 ± 0.42	0.996
Platelet (x 10^3/μl)	257 ± 77	256 ± 66	229 ± 52	250 ± 64	248 ± 62	245 ± 58	0.924
White blood cell (x 10^3/μl)	5.69 ± 1.27	6.22 ± 1.25	5.17 ± 1.40	6.20 ± 1.48	5.30 ± 1.66	6.50 ± 1.63	0.098
Neutrophil (x 10^3/μl)	2.98 ± 0.81	3.39 ± 0.80	2.65 ± 0.94	3.35 ± 1.04	2.74 ± 1.17	3.58 ± 1.19	0.056
Lymphocyte (x 10^3/μl)	1.98 ± 0.52	2.08 ± 0.45	1.83 ± 0.48	2.13 ± 0.46	1.88 ± 0.52	2.12 ± 0.46	0.585
Monocyte (x 10^3/μl)	0.47 ± 0.16	0.48 ± 0.14	0.40 ± 0.13	0.47 ± 0.15	0.44 ± 0.17	0.53 ± 0.18	0.328
Eosinophil (x 10^3/μl)	0.24 ± 0.17	0.23 ± 0.14	0.25 ± 0.19	0.25 ± 0.18	0.21 ± 0.14	0.24 ± 0.15	0.993
Basophil (x 10^3/μl)	0.03 ± 0.05	0.02 ± 0.04	0.02 ± 0.04	0.02 ± 0.04	0.03 ± 0.05	0.01 ± 0.03	0.574

Supplemental Material, Table S3. Comparison of nCounter data between delta-FAP and delta-DEP (two-sided test). Positive log2 fold change indicates that expression increased more after DEP that it did after FAP.

miRNA	log2 fold change	t	p value	FDR
hsa-miR-320a	-0.68	-6.28	0.000014	0.0011
hsa-let-7d	0.30	3.11	0.007	0.28
hsa-miR-215	0.34	2.79	0.014	0.37
hsa-miR-22	-0.26	-2.62	0.019	0.39
hsa-miR-937	-0.19	-2.46	0.026	0.39
hsa-miR-24	0.17	2.42	0.029	0.39
hsa-miR-29a	0.47	2.15	0.048	0.54

Supplemental Material, Table S4. Comparison of nCounter data between post-FAP and post-DEP (two-sided test). Positive log2 fold change indicates higher expression after DEP relative to FAP.

miRNA	log2 fold change	log2 average code count	t	p value	FDR
hsa-miR-21	0.36	8.72	4.93	0.0001	0.011
hsa-miR-144	0.56	9.42	4.48	0.0004	0.014
hsa-miR-30e	0.46	6.74	4.10	0.0008	0.021
hsa-miR-215	0.37	5.26	3.62	0.0022	0.044
hsa-miR-148a	0.37	6.95	3.34	0.0040	0.063
hsa-miR-15a	0.23	11.43	3.27	0.0047	0.063
hsa-miR-183	0.31	7.64	3.16	0.0058	0.068
hsa-miR-125a-5p	-0.24	6.77	-2.68	0.016	0.16
hsa-miR-181a	0.28	9.06	2.49	0.024	0.16
hsa-miR-29a	0.35	5.69	2.48	0.024	0.16
hsa-miR-92b	0.14	10.20	2.45	0.026	0.16
hsa-miR-25	0.15	14.74	2.44	0.027	0.16
hsa-miR-937	-0.22	6.35	-2.44	0.027	0.16
hsa-miR-148b	0.18	8.45	2.41	0.028	0.16
hsa-miR-142-3p	0.37	10.75	2.35	0.032	0.17
ebv-miR-BHRF1-1	0.12	9.50	2.18	0.044	0.22
hsa-miR-1274b	-0.13	7.04	-2.15	0.047	0.22

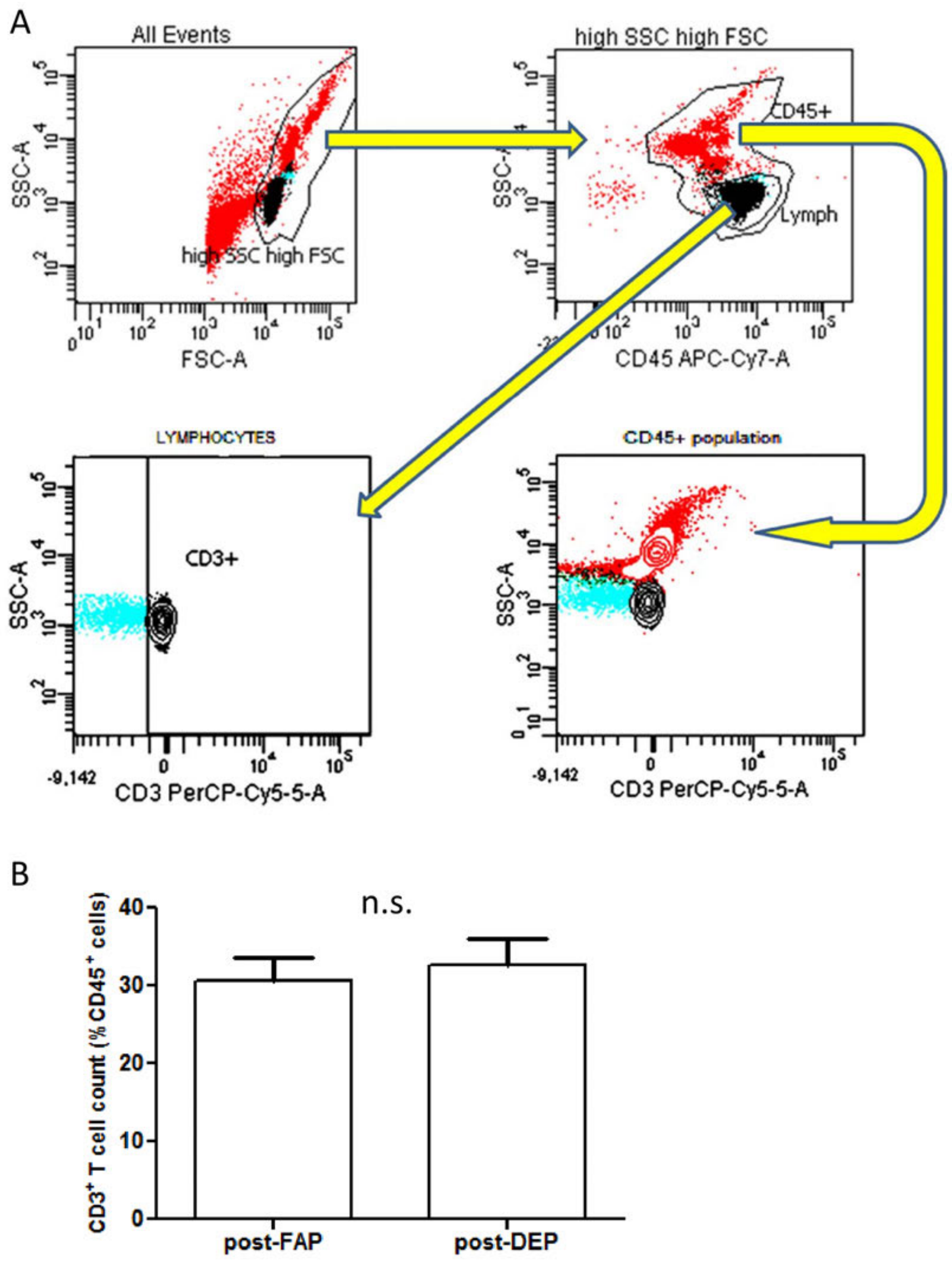

Supplemental Material, Figure S1. CD3 positive T cell counts. A. analytical procedure in flow cytometry (described in Supplemental Material, Methods). B. Percentage of $CD3^+$ T lymphocytes out of the $CD45^+$ cells (leukocytes) comparing post DEP and FAP. n.s.: not significant

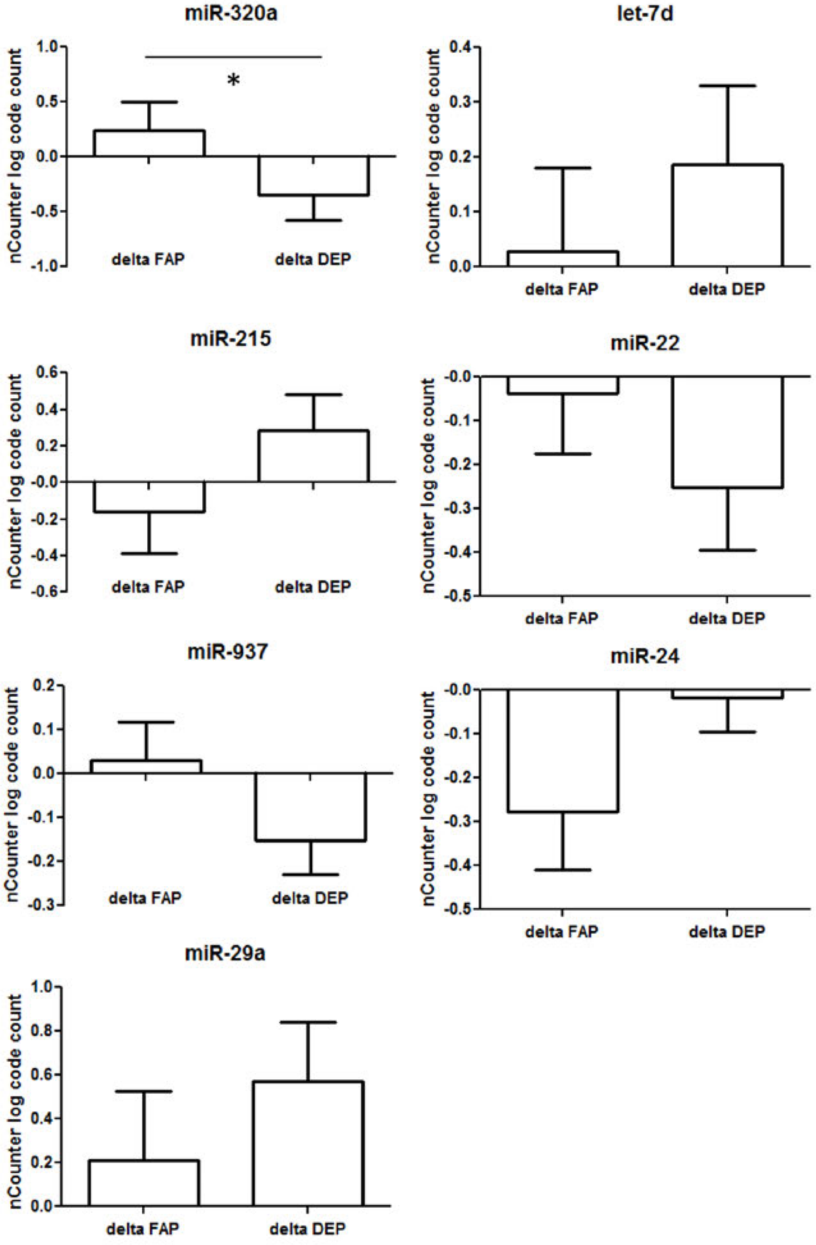

Supplemental Material, Figure S2. Comparison of nCounter data between delta-FAP and delta-DEP. Log2 transformed nCounter data for delta-FAP and delta-DEP are shown. Bars represent mean and error bars represent standard error of the mean. *: FDR <0.05

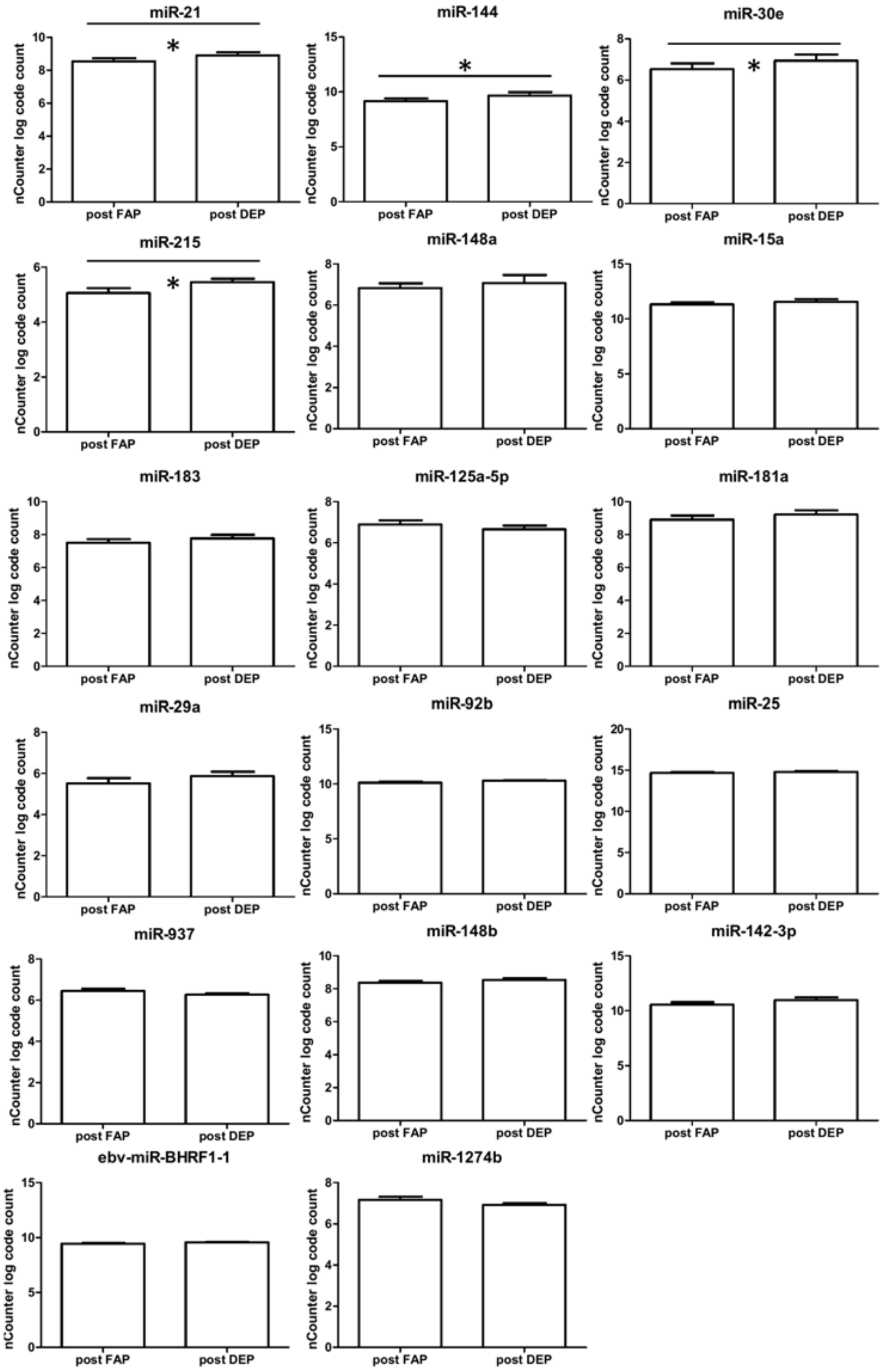

Supplemental Material, Figure S3. Comparison of nCounter data between post-FAP and post-DEP. Log2 transformed nCounter data for post-FAP and post-DEP are shown. Bars represent mean and error bars represent standard error of the mean. *: FDR <0.05

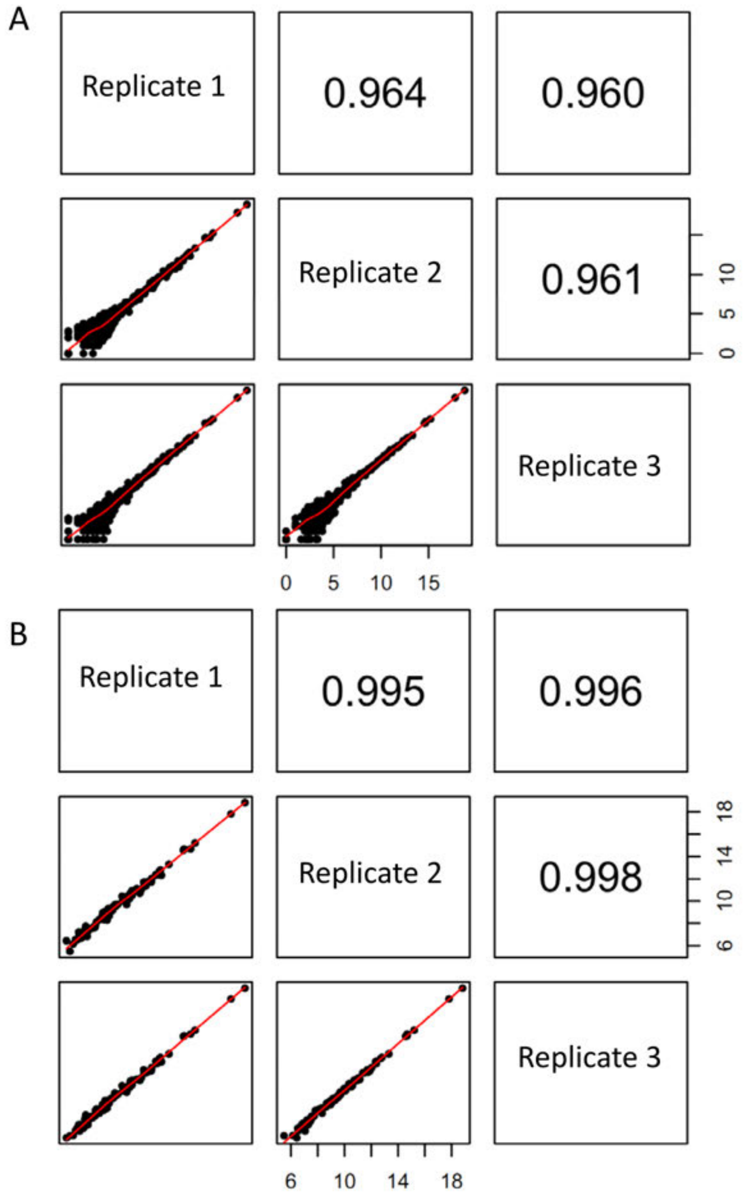

Supplemental Material, Figure S4. Scatter plots of technical replicates for nCounter assay. Correlation of miRNA data from representative technical replicate (triplicate) experiment is shown. Each axis represents log2 code counts for each miRNA. Pearson's correlation coefficients are shown in the upper right panels. A: Unnormalized code count for 734 miRNAs. B: Normalized code count for analysed 81 miRNAs.

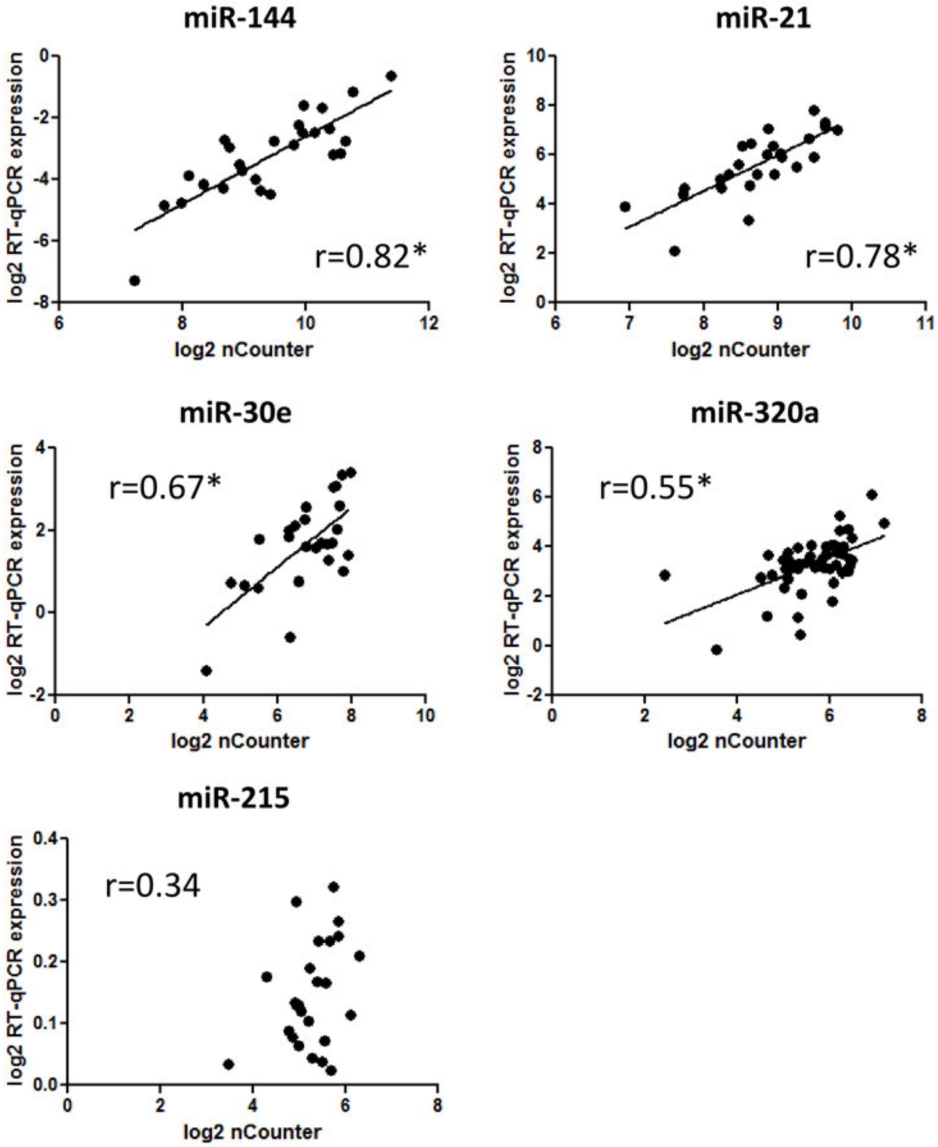

Supplemental Material, Figure S5. Scatter plots of candidate miRNAs comparing nCounter assay and RT-qPCR. Correlations of miRNA expression (miR-144, miR-21, miR-30e, miR-320a, miR-215) between NanoString nCounter assay and RT-qPCR are shown. X and Y axes show log2 transformed normalized nCounter code count data and RT-qPCR expression levels, respectively. The expression level was normalized with sum of miRNA counts in nCounter data, and with RNU44 levels in RT-qPCR data. r: Pearson's correlation coefficients, *: p value <0.05

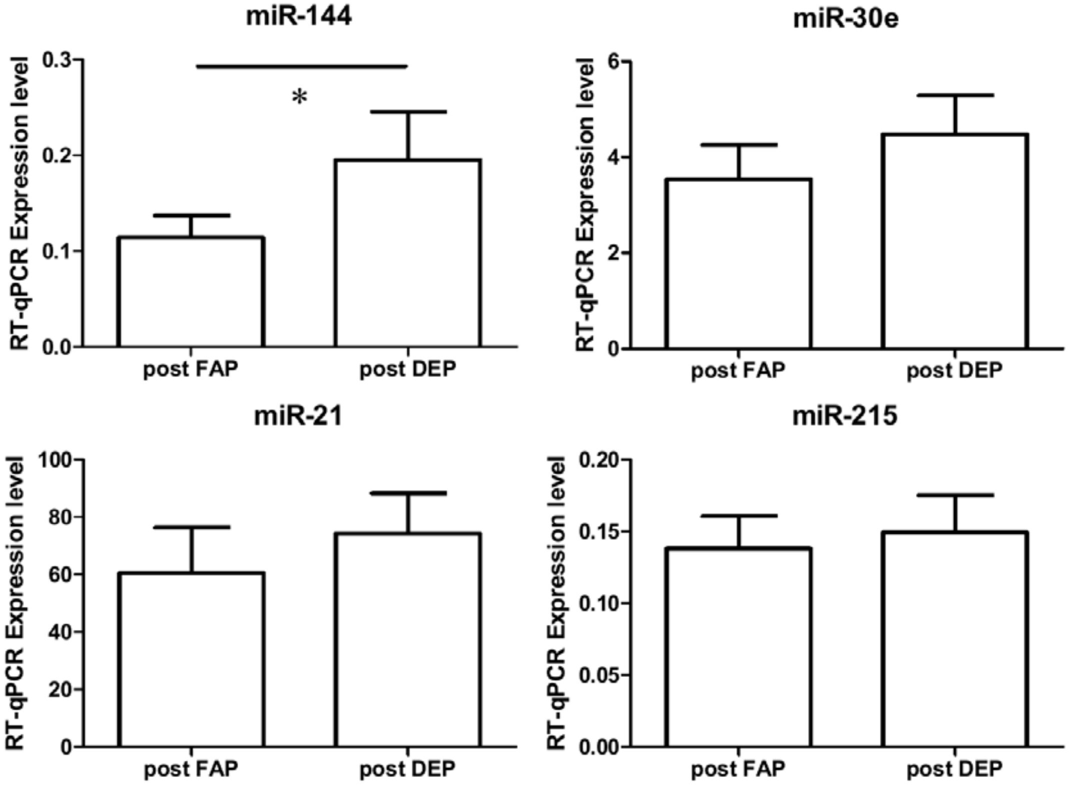

Supplemental Material, Figure S6. Validation of miRNA levels with RT-qPCR between post-FAP and post-DEP. Relative expression levels measured by RT-qPCR for post-FAP and post-DEP are shown. Bars represent mean and error bars represent standard error of the mean. *: FDR <0.05

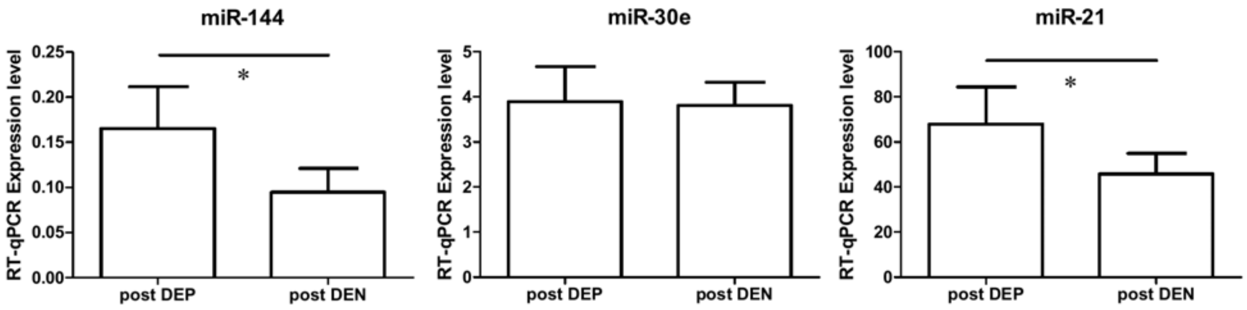

Supplemental Material, Figure S7. Comparison of miRNA level between post-DEP and post-DEN. Relative expression levels measured by RT-qPCR for post-DEP and post-DEN are shown. Bars represent mean and error bars represent standard error of the mean. *: FDR <0.05
